# Low thyroid hormone levels improve survival in murine model for ocular melanoma

**DOI:** 10.18632/oncotarget.3566

**Published:** 2015-03-14

**Authors:** Ido Didi Fabian, Mordechai Rosner, Ina Fabian, Vicktoria Vishnevskia-Dai, Ofira Zloto, Elena Shinderman Maman, Keren Cohen, Martin Ellis, Hung-Yun Lin, Aleck Hercbergs, Paul J. Davis, Osnat Ashur-Fabian

**Affiliations:** ^1^ Goldschleger Eye Institute, Sheba Medical Center, Tel Hashomer, affiliated to Sackler Faculty of Medicine, Tel Aviv University, Tel Aviv, Israel; ^2^ Department of Cell and Developmental Biology, the Sackler Faculty of Medicine, Tel Aviv University, Tel Aviv, Israel; ^3^ Department of Human Molecular Genetics and Biochemistry, the Sackler Faculty of Medicine, Tel Aviv University, Tel Aviv, Israel; ^4^ Translational Hemato-Oncology Laboratory, The Hematology Institute and Blood Bank, Meir Medical Center, Kfar-Saba, affiliated to Sackler Faculty of Medicine, Tel Aviv University, Tel Aviv, Israel; ^5^ Institute of Cancer Biology and Drug Discovery, School of Medical Technology, Taipei Medical University, Taipei, Taiwan; ^6^ Pharmaceutical Research Institute, Albany College of Pharmacy and Health Sciences, Albany, NY, USA; ^7^ Department of Radiation Oncology, Cleveland Clinic, Cleveland, OH, USA; ^8^ Department of Medicine, Albany Medical College, Albany, NY, USA

**Keywords:** integrin, uveal melanoma, thyroid

## Abstract

Uveal melanoma is highly metastatic, prognosis is poor and there are no effective treatments to extend survival. Accumulating evidence suggests that thyroid hormones have a mitogenic effect via binding to αvβ3 integrin. We aimed to examine the impact of thyroid status on survival in a murine B16F10 model for ocular melanoma, highly expressing the integrin. In two independent experiments oral propylthiouracil (PTU) was used to induce hypothyroidism (n=9), thyroxine to induce hyperthyroidism (n=11) and mice given plain water served as control (n=8). At day 21, the subretinal space was inoculated with 10^2^ B16F10 cells. In non-inoculated mice (n=6 of each group) serum free T_4_ (FT_4_) levels were measured and additional non-inoculated mice (3 given PTU and 4 given thyroxine or water) served as internal control to demonstrate the impact of the dissolved substance. The PTU-inoculated mice showed clinical evidence of intraocular tumor growth significantly later than the thyroxine mice (*P*=0.003) and survival time was significantly longer (*P*<0.001). FT_4_ levels differed significantly between groups (*P*<0.001) and with no signs of illness in the internal control group. Our findings suggest that hyperthyroidism shortens survival, whereas relative hypothyroidism may have a protective role in metastatic ocular melanoma.

## INTRODUCTION

Uveal melanoma (UM) is the most common primary intraocular malignancy in adults, and a condition that may lead to fatal metastases [1, 2]. It is estimated that 50% of UM patients may develop metastatic disease, with the spread of tumor cells being predominantly to the liver [3, 4]. Metastatic UM has a poor prognosis, with a reported median survival of 4-15 months [5]. Despite considerable improvement in local treatments, especially that of eye-preserving radiotherapy, there has been no change in survival rates in the past 4 decades [4, 6]. Taken together, these poor outcomes emphasize the need for alternatives to traditional treatments for UM.

For more than two decades, the actions of thyroid hormones in a variety of tumor models have been described to influence proliferation and angiogenesis in breast, glial, thyroid and lung cancer cells [7]. In 2003, the pharmacological induction of mild biochemical hypothyroidism by means of propylthiouracil (PTU) significantly improved survival duration in patients with glioblastoma multiforme, one of the most aggressive forms of brain tumors [8]. In addition, hypothyroidism was found to increase response rates to chemotherapy and radiation therapy in preclinical models and clinical studies of various tumor types (reviewed in [9] and [10]). In contrast, the hyperthyroid state was found to be associated with increased cancer risk in a prospective population study [11]. The possible clinical roles of thyroid hormone in cancer behavior have recently been reviewed [12]. One explanation for the possible association between thyroid hormone levels and cancer was recently given in the form of a receptor for L-thyroxine (T_4_) and 3,5,3′-triiodo-L-thyronine (T_3_) on plasma membrane integrin αvβ3 [10, 13]. This integrin appears to mediate the proliferative action of the hormone on blood vessel cells and on tumor cells [10]

To the best of our knowledge, the influence of thyroid hormone on UM has not been investigated to date. We therefore determined the impact of thyroid status on survival of C57BL/6 mice inoculated with B16F10 melanoma cells and confirmed that hyperthyroidism shortened survival in metastatic ocular melanoma, while hypothyroidism had a significant protective effect.

## RESULTS

**Figure 1:**
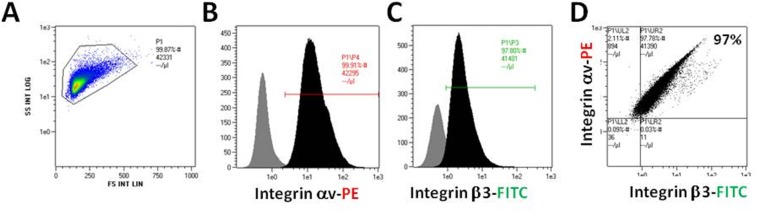
B16F10 cells were collected and measured for αv/β3 monomers expression by flow cytometry (A) All cells, (B) integrin αv monomer-positive cells, (C) integrin β3 positive cells and (D) αvβ3 positive cells.

For *in vivo* testing we used the mouse melanoma B16F10 cell line, given its ability to form tumors when injected into the anterior eye chamber of murine eyes [14-17]. Of the 100,000 B16F10 melanoma cells that were evaluated (Figure 1A), a high expression of αv (Figure 1B) and β3 monomers (Figure 1C) was documented. All melanoma cells were positive for both αv and β3 monomers, thus demonstrating high expressions of the αvβ3 dimer on the B16F10 cell membrane (Figure 1D). These results indicated that B16F10 melanoma cells were a valid platform to examine *in vivo* the effect of the thyroid status via the αvβ3 integrin.

**Figure 2:**
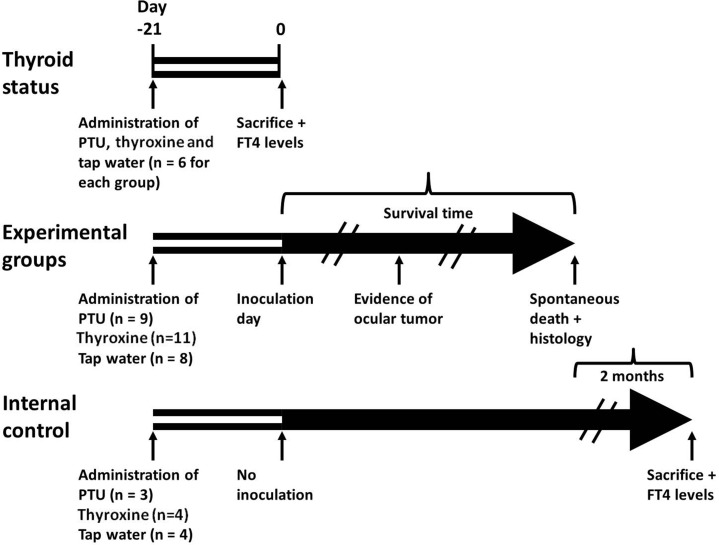
Treatment schedules for the study groups Forty six mice were given propylthiouracil (PTU), thyroxine, both in drinking water, or tap water (control) for 21 days. Six mice from each group were then removed in order to measure FT_4_ levels (Thyroid status), after which they were sacrificed. Of the remaining 28 mice—9 given PTU, 11 given thyroxine and 8 given tap water (Experimental groups)—were inoculated with tumor cells (day 0) and monitored daily, and first sign of intraocular tumor growth was recorded. The time from inoculation to spontaneous death was defined as the survival time. The internal control consisted of 11 additional mice, 3 given PTU, 4 given thyroxine and 4 given tap water; they were not inoculated with tumor cells. These mice were followed-up and sacrificed 2 months after the last experimental mouse had died (blood samples had been obtained for FT_4_ level determination beforehand).

Figure 2 demonstrates the treatment schedules used in this study, which consisted of two identical independent experiments. A hypothyroid state was induced in 9 mice by adding PTU to the drinking water and a hyperthyroid state was induced in 11 mice by adding thyroxine to drinking water. Eight additional mice were given plain tap water and served as controls. Six mice of each group before inoculation represented the thyroid status of their specified group by means of measuring the serum free T_4_ levels (FT_4_). FT_4_ levels in the PTU hypothyroid group (7.7 ± 0.4 pmol/L) and the thyroxine hyperthyroid group (43.7 ± 1.8 pmol/L) were significantly different from those of the controls (27.5 ± 2.3 pmol/L) (*p*<0.001).

**Figure 3:**
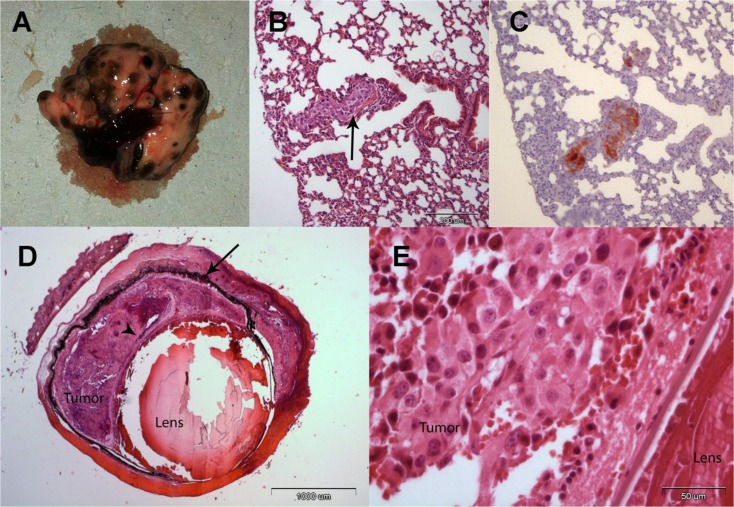
(A) Macro metastasis of B16F10 cells in the lung of a representative mice. (B) An aggregate of large epithelioid melanoma cells with expanded cytoplasm, large nuclei and prominent nucleoli within it (arrow), is surrounded by typical lung tissue (H&EX10). (C) An adjacent section of the lung is positive for S100 immunostaining confirming the presence of melanoma cells (X10). (D) Enucleated murine eye showing the intraocular tumor located behind the lens, between the pigment epithelium (arrow) and retina (arrowhead, H&EX2). (E) Tumor cells behind the lens (H&EX40).

After 21 days (Figure 2, experimental groups, day 0), the subretinal space of each mouse's right eye was inoculated with aliquots of 10^2^ B16F10 cells/1 μL PBS using a transconjunctival approach as previously described [18]. There were no cases of cell reflux following tumor inoculation and the subconjunctival space remained free of tumor cells. As expected from preliminary experiments (data not shown), tumors were evident about two weeks from inoculation. Enucleated eyes and dissected lungs of 2 mice from each experimental group were sent for pathological processing and H&E and S-100 staining. Macro metastasis of B16F10 cells (Figure 3A), surrounded by typical lung tissue, were identified in the lung (Figure 3B). Positive S100 immunostaining confirmed the presence of melanoma cells (Figure 3C). Typical melanoma cells were also detected behind the lens, between the pigment epithelium and retina (Figure 3D and 3E) in all of the inoculated eyes specimens.

**Figure 4:**
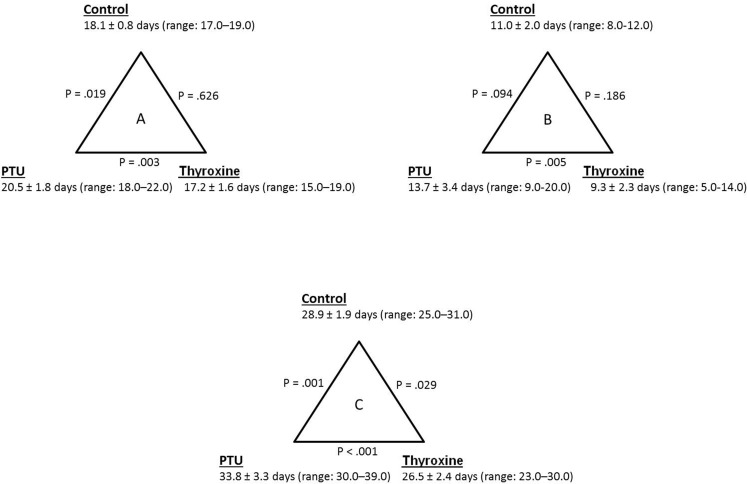
(A) Differences in time from inoculation to clinical evidence of intraocular tumor growth, (B) tumor growth to death and (C) inoculation to death (survival time) between the PTU (n=8), thyroxine (n=11) and control (n=9) groups. Data are presented as mean ± standard deviation. PTU – propylthiouracil.

Figure 4 shows the differences in time from inoculation to clinical evidence of intraocular tumor growth (Figure 4A), tumor growth to death (Figure 4B) and the survival time from the day of inoculation till death (Figure 4C) of the different experimental groups. The PTU group had a significantly longer inoculation-to-tumor time (Figure 4A) than the control group (*p* = 0.019), whereas there were no significant differences between the control and thyroxine groups (*p* = 0.626). Of note, intraocular tumors were diagnosed significantly earlier in the thyroxine group compared to the PTU group (*p* = 0.003).

When assessing the tumor-to-death time (Figure 4B), the PTU group exhibited a significantly longer period compared to the thyroxine group (*p* = 0.005). Interestingly, no significant differences were observed between the PTU and thyroxine groups compared to the control group (*p* ≥ 0.094).

**Figure 5:**
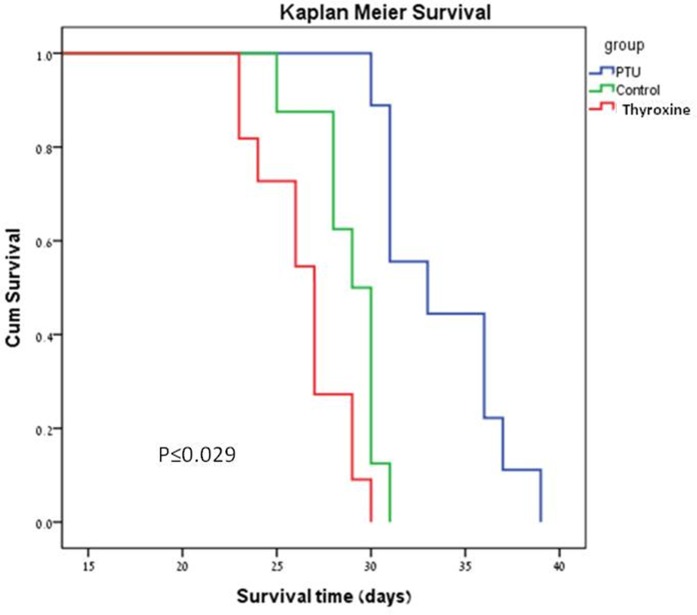
Kaplan-Meier survival plots of the PTU, FT (thyroxine) and control groups

Finally, the average survival for all groups was 29.5 ± 4.0 days (range: 23.0–39.0 days). There were significant differences in survival between groups (Figures 4C and 5). The PTU group survived significantly longer (33.8 ± 3.3 days, *p* = 0.001) than the control group (28.9 ± 1.9 days), the latter representing the natural history of a melanoma-tumor bearing murine eye. The addition of thyroxine to drinking water significantly shortened survival (26.5 ± 2.4 days, *p* = 0.029) compared to the controls.

An internal control group served to verify that the inoculated mice died of the tumor and not as a result of the thyroid status. This group was comprised of 3 PTU mice and 4 mice each of the thyroxine and control groups which were not inoculated with tumor cells. These mice were followed daily for signs of illness, which were not detected at any stage. Two months after the last experimental mouse had spontaneously died blood samples were obtained from the internal control group mice for determining their FT_4_ levels, after which they were sacrificed. The FT4 levels for the PTU, thyroxine and control groups were 6.9 ± 0.2 pmol/L, 42.1 ± 3.4 pmol/L and 23.4 ± 0.6 pmol/L (p < 0.001). As noted above, serum FT_4_ values in the experimental groups were appropriately different from measurements in the control animals.

**Figure 6:**
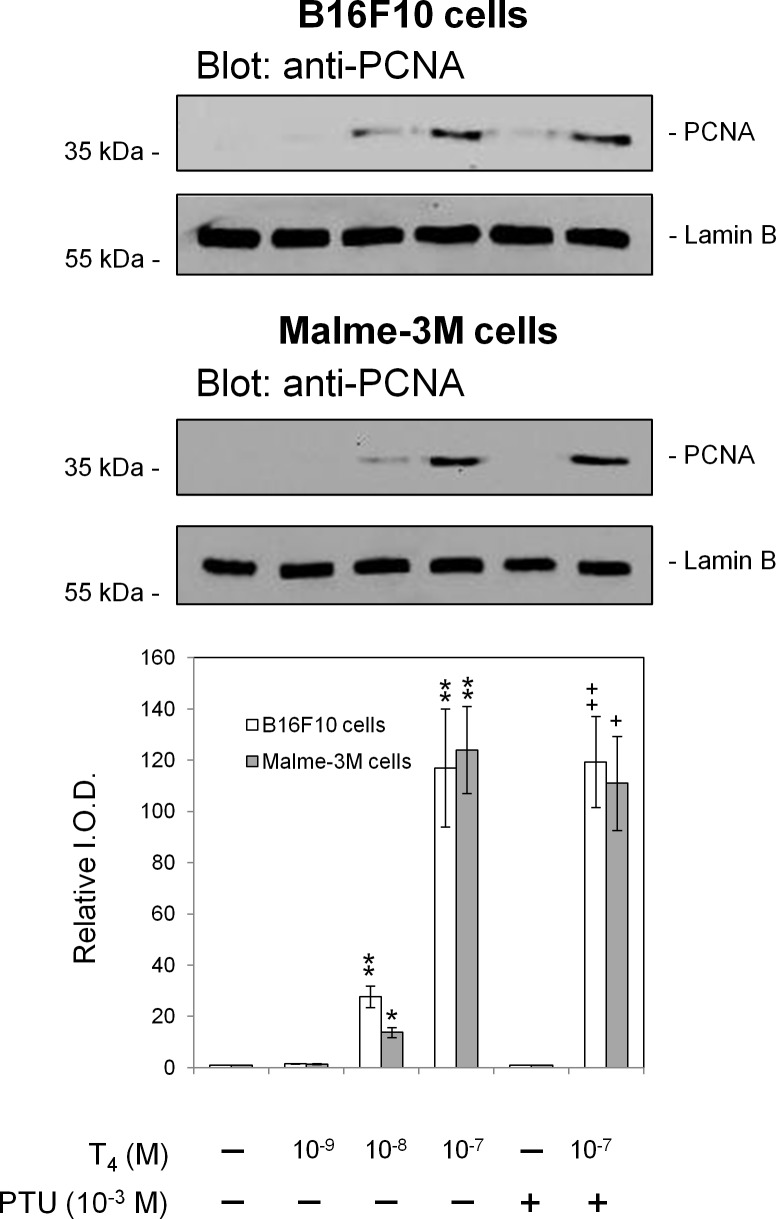
Proliferative effects of T on mouse melanoma (B16F10) and human melanoma (Malme-3M) cells *in vitro* In this set of representative PCNA immunoblots, T_4_ at 10^−8^ and 10^−7^ M is shown to stimulate tumor cell proliferation. At 10^−7^ M in the buffer system used, T_4_ generates a physiologic free T_4_ concentration [13]. PTU treatment of cells did not affect the proliferation action of T_4_ indicating that conversion of T_4_ to T_3_ is not involved in this tumor cell effect of the hormone. Proliferative actions of unmodified T_4_ are expressed via integrin αvβ3 [27, 28]. Independent experiments: B16F10 cells, n = 3; Malme-3M cells, n = 2; *p < 0.05, ** p <0.01 by *t* test, comparison with vehicle solvent control; ^+^p < 0.05, ^++^p < 0.01, comparison with PTU, alone. Lamin B served as a loading control.

Figure 6 depicts the proliferative action of T_4_ *in vitro* on B16F10 melanoma cells and on a human melanoma cell line, Malme-3M, thus indicating effects of thyroid hormone in our *in vivo* studies are not limited to B16F10 cells. The total T_4_ concentration of 10^−7^ M achieves a physiological FT_4_ level of <10^−10^ M in the culture medium used [13]. Propylthioracil inhibits cell conversion of T_4_ to T_3_ and did not inhibit the proliferative action of T_4_ on these cell lines; thus, the proliferative response of the melanoma cells to T_4_ *in vitro* reflects action of the latter as a hormone, not as a prohormone source of T_3_.

## DISCUSSION

The prognosis for patients with UM is poor and the mortality rate remains high, due to development of metastatic disease which is highly resistant to systemic therapy. A recent and comprehensive review of the systemic treatment of metastatic UM concluded that the limited efficacy of current treatment approaches underscores the medical need for more effective treatment options [19]. Accumulating data suggest that hyperthyroidism may increase the risk of certain non-ocular solid tumors, whereas hypothyroidism may delay onset and reduce aggressiveness of cancers [9, 12]. Based on these data, we designed a set of experiments to examine the impact of different levels of circulating FT_4_ on survival duration in a UM murine model. The recent discovery of a receptor on plasma membrane integrin αvβ3 which may mediate the proliferative action of thyroid hormone on tumor cells [10], motivated us to examine its expression on B16F10 melanoma cells, which were chosen for the present study. Our results documented, similarly to previous reports [20] that these cells were positive for both αv and β3 integrin monomers, indicating that B16F10 melanoma cells were a valid platform for our *in vivo* assays. Our hypothesis that a hyperthyroid state will shorten survival and that a hypothyroid state will prolong life in an ocular melanoma murine model was confirmed, in support of the body of evidence showing the association of thyroid hormone levels and cancer [9, 12].

It has been established that the B16F10 cell line metastasizes predominantly to the lungs [21] and this was confirmed in all of the animals sampled in our study, implying that it was the tumors' systemic spread that killed the mice. Additional support came from our internal control group that unambiguously showed that the mice died of the tumor and not as a result of their unbalanced thyroid status. Finally, Theodossiou and co-workers had shown that tumor responses are a result of the hypothyroid state rather than PTU in a lung and prostate cancer murine model, and our results in a UM murine model are in agreement with theirs [22].

Analysis of the inoculation-to-tumor and tumor-to-death time frames revealed similar trends not only for the metastatic disease, but also for the impact of thyroid hormone levels on the intraocular tumor. These results were best reflected in the significant differences between the PTU and thyroxine groups, which represent the two ends of the thyroid function spectrum. It is possible that inclusion of larger numbers of mice in the current study would have resulted in even more significant differences, compared to controls. One possible implication of these results is that it would appear to be worthwhile to combine the beneficial impact of the hypothyroid state with local treatment modalities to the intraocular tumor. We believe that further investigation of this concept has considerable merit.

In a literature search of tumor cells inoculation we found that in all studies around 10^5^ cells (range: 1–5 × 10^5^ cells) were injected into the posterior or anterior chambers [14, 16, 17, 23, 24]. When amounts of cells of this size were used in preliminary studies, the eyes burst at about one week after inoculation, limiting the chance for a therapeutic effect. We therefore diluted the number of cells to be inoculated in our study to 10^2^ cells/1 μL, and an inoculum of this size was found to provide a reliable, reproducible and, importantly, effective model that simulated the natural course of the disease.

Since the mitogenic effects of thyroid hormones presented in this work are mediated via the αvβ3 integrin, we searched the literature for its presence on human primary UM cells, and found that it is expressed in all tumor subtypes, including spindle, epithelioid and mixed cell tumors [25], suggesting the testing of the integrin expression as a therapeutic approach. Nowadays, in many ocular oncology centers intraocular tumor biopsies for prognostic purposes are considered common practice [26]. The tissue obtained may serve to evaluate the integrin abundance on tumor cells as well, and, if present, the patient may be a candidate for treatment. The evidence that supports mediation by αvβ3 of the thyroid hormone effect in the current study is incomplete, but includes several important observations. The B16F10 cells express the integrin. The animals were treated with PTU, an agent that blocks T_4_ to T_3_ conversion, and thus the critical intracellular thyroid hormone, T_3_, could not be generated. T_4_ in physiological concentrations is a hormone, rather than a prohormone, at the cell surface thyroid hormone receptor on αvβ3; in a variety of tumor cell model systems, T_4_ has been shown to be a proliferative agent via αvβ3 and is the primary ligand of the integrin [27]. *In vitro* cell culture studies here confirmed in two human melanoma cells that T_4_ in the presence of PTU indeed supported tumor cell proliferation.

This study has several limitations. As opposed to the clear-cut time points of inoculation and death, detecting the beginnings of intraocular tumor growth may be subject to error. However, it was performed in the same manner for all the experimental groups, thereby excluding a potential bias. Although the numbers of animals were large enough in order to obtain significant survival differences between the experimental groups, splitting these intervals into before and after signs of ocular tumor growth were evident, affected the statistical power, resulting in mixed results (Figure 4). Nonetheless, trends of the impact of thyroid status could clearly be identified in these cases, as well. Substantial strength of the present study lies in the fact that it is comprised of two identical and independent experiments, both of which led to similar results.

Intraocular inoculated B16 melanoma cells, although derived from cutaneous melanoma, are a good platform and commonly used to model ocular melanoma, including for the evaluation of novel therapeutic approaches [14, 16, 17, 23, 24]. Although uveal and cutaneous melanoma cells differ biologically, yet, in the current study a proof of concept of the impact of the thyroid status on intraocular melanoma that metastasizes to a distant site and eventually results in death, was achieved. We are currently testing various therapeutic modalities (not published) using a cutaneous melanoma cell line that metastasizes predominantly to the liver, mimicking UM metastatic spread. In the present experiments, B16F10 cells only were used in the animal models, but our cell culture studies confirmed existence of the proliferative effect of thyroid hormone (T_4_) in both B16F10 and Malme-3M melanoma cells.

Finally, a substantial number of UM patients develop metastatic disease with attendant poor prognosis and for which there are no effective treatments. Our study results indicate that survival in an ocular melanoma murine model is extended due to induction of the hypothyroid state and shortened due to a hyperthyroid state, in support of a growing body of knowledge that supports an association between thyroid hormone levels and cancer cell proliferation. These findings and the beneficial effect of the hypothyroid state on the primary tumor suggest that there also might be therapeutic benefit in the induction of mild biochemical hypothyroidism [12] in this tumor setting or, alternatively, of administering novel integrin αvβ3-targeted therapies [27, 28] as soon as the primary uveal tumor is diagnosed. Further *in vivo* investigations are warranted to substantiate the impact of thyroid hormone status on ocular melanoma.

## MATERIALS AND METHODS

### Cell lines and expression of integrin αvβ3

B16F10 mouse melanoma cell line (ATCC, CRL-6475) was cultured in RPMI 1640 medium, supplemented with 10% (v/v) heat inactivated fetal calf serum, 2 mM L-glutamine and antibiotics (penicillin/streptomycin), in a humidified atmosphere of 5% CO2 at 37^0^C. Given that the αvβ3 integrin contains a receptor site for T_3_ and T_4_, we measured the expression of this integrin on B16F10 melanoma cells which were utilized in the animal models. B16F10 melanoma cells (100,000 cells) were harvested and labeled with 50 μg/ml PE-αv antibodies (Clone RMV-7, Abcam), and FITC-β3 antibodies (Clone HM beta 3.1, Abcam) in 100 mL phosphate-buffered saline (PBS). Following incubation for 15 minutes at room temperature, the cells were centrifuged, diluted in PBS and analyzed by a fluorescence-activated cell sorter (FACS, Navios Flow Cytometer; Beckman Coulter, Inc). For *in* vitro studies, Malme-3M cells (ATCC HTB-64) and B16F10 cells were cultured in DMEM, supplemented with 10% hormone-stripped FBS [13]. L-T4 and propylthiouracil for these studies were obtained from Sigma-Aldrich. Treatment of harvested cells and western blotting for proliferating cell nuclear antigen (PCNA) were by our previously reported methods [13].

### Animals

Study animals were wild-type male C57BL/6 mice aged 8 weeks (Harlan Laboratories Ltd, Ein Kerem, Jerusalem). The mice were acclimated to our vivarium for 1 week prior to their use according to study protocols. Up to 6 animals were housed in a cage under conventional conditions and fed chow and water *ad libitum*. All animal procedures and experiments were conducted with approval and under the supervision of the Institutional Animal Care Committee at Tel-Aviv University, and conformed to recommendations of the Association for Research in Vision and Ophthalmology Statement for the Use of Animals in Ophthalmic and Vision Research.

### Experimental groups and inoculation of tumor cells

Figure 2 demonstrates the treatment schedules used in this study, which consisted of two identical independent experiments. A hypothyroid state was induced in 9 mice by adding PTU (Sigma-Aldrich Israel Ltd. Rehovot, Israel, 1 mg/mL) to their drinking water and a hyperthyroid state was induced in 11 mice by adding thyroxine (Sigma-Aldrich Israel Ltd., 6 μg/mL) to drinking water. Eight additional mice were given plain tap water and served as controls. After 21 days (Figure 2, experimental groups, day 0), the subretinal space of each mouse's right eye was inoculated with aliquots of 10^2^ B16F10 cells/1 μL PBS using a transconjunctival approach as previously described [18]. Mice were anesthetized with a mixture of ketamine and xylazine (120 mg/kg ketamine, 10 mg/kg xylazine), and the experimental eye was desensitized by a drop of oxybuprocaine (Dr. Fischer, Bnei Barak, Israel). Under a dissecting microscope, a 30 gauge needle was inserted approximately 1 mm posterior to the limbus through the conjunctiva and sclera and into the subretinal space. The tip of a 10 μL glass syringe with a 32 gauge blunt needle (Hamilton Co., Bonaduz, Switzerland) was introduced into the subretinal space via the needle track, and a 1 μL suspension of tumor cells was then injected into the eyes of the animals.

### Clinical follow-up and study definitions

All of the mice were checked daily for clinical evidence of intraocular tumor growth. These signs appeared in the form of intraocular bleeding, turbidity, or both in preliminary experiments (data not shown). When any of these signs became evident, the mouse was transferred to a separate cage and followed-up until death. The interval between inoculation of tumor cells and death was defined as the survival time. The interval between inoculation of tumor cells and first clinical evidence of intraocular tumor growth was defined as the inoculation-to-tumor time, and the interval between first clinical evidence of intraocular tumor growth to death was defined as the tumor-to-death time. All of these data was recorded and analyzed.

### Free T4 assay

Eighteen additional non-inoculated mice were treated with PTU, thyroxine or plain water (n=6 in each group) for 21 days and their thyroid function was assessed (Figure 2, Thyroid status, day 0). Thyroid function was measured by obtaining blood samples with a small glass capillary tube positioned behind the eye in the ophthalmic venous plexus. Serum free T_4_ (FT_4_) levels were determined using a FT_4_ radioimmunoassay kit (Beckman Coulter Oslo, Norway), after which the mice were sacrificed.

### Internal control group

Eleven additional non-inoculated mice (Figure 2, Internal control) were given drinking water containing PTU (n=3), thyroxine (n=4) or tap water (n=4). These mice served as internal control in each of the study groups in order to determine the systemic impact of the dissolved substance. They were followed-up on a daily basis and sacrificed 2 months after the last mouse of the experimental group had died (blood samples had been obtained for FT_4_ level determination beforehand).

### Histopathological and immunohistochemical studies

The tumor-bearing eyes of all the inoculated mice and lungs of a sample of 2 mice in each experimental group were harvested and sent for pathological and immunohistochemical evaluations. Formalin-fixed, paraffin-embedded sections of the collected specimens were hematoxylin and eosin (H&E) stained for histopathologic assessment. For immunostaining, the slides were warmed to 60^0^C for 60 minutes, dewaxed in xylene and rehydrated. Hydrogen peroxide (H_2_O_2_, 3% in PBS) was used to block endogenous peroxidase activity. After being rinsed in PBS, the sections were incubated for 60 minutes at room temperature with anti-S100 (Z0311, 1:1000, Dako, Herzliya, Israel), a melanoma marker. Detection was performed with Envision+ System-HRP Labelled Polymer Anti-Rabbit (K4003, Dako). The binding antibody was visualized with chromogen AEC substrate (Invitrogen Corporation). Sections were counterstained with hematoxylin and cover-slipped with an aqueous mounting fluid (Glycerol, Dako). The stained sections were reviewed with a light microscope and analyzed by a pathologist.

### Statistics

The results were expressed as the mean ± SD and *P* < 0.05 was considered statistically significant. The Log Rank (Mantel-Cox) test was used to compare the survival data, the inoculation–tumor time and the tumor–death time between groups. A Kaplan-Meier plot was generated graphically to demonstrate the survival curves of the experimental groups. Group differences in FT_4_ values were analyzed using post hoc Tukey test one-way ANOVA. Analysis of the *in vitro* cell proliferation studies was by Student's *t* test.
